# Enhanced deep learning model for precise nodule localization and recurrence risk prediction following curative-intent surgery for lung cancer

**DOI:** 10.1371/journal.pone.0300442

**Published:** 2024-07-12

**Authors:** Jihwan Park, Mi Jung Rho, Mi Hyoung Moon

**Affiliations:** 1 College of Liberal Arts, Dankook University, Cheonan-si, Chungcheongnam-do, Republic of Korea; 2 College of Health Science, Dankook University, Cheonan-si, Chungcheongnam-do, Republic of Korea; 3 Department of Thoracic and Cardiovascular Surgery, Seoul St. Mary’s Hospital, College of Medicine, The Catholic University of Korea, Seoul, Republic of Korea; University of Illinois Urbana-Champaign, UNITED STATES OF AMERICA

## Abstract

**Purpose:**

Radical surgery is the primary treatment for early-stage resectable lung cancer, yet recurrence after curative surgery is not uncommon. Identifying patients at high risk of recurrence using preoperative computed tomography (CT) images could enable more aggressive surgical approaches, shorter surveillance intervals, and intensified adjuvant treatments. This study aims to analyze lung cancer sites in CT images to predict potential recurrences in high-risk individuals.

**Methods:**

We retrieved anonymized imaging and clinical data from an institutional database, focusing on patients who underwent curative pulmonary resections for non-small cell lung cancers. Our study used a deep learning model, the Mask Region-based Convolutional Neural Network (MRCNN), to predict cancer locations and assign recurrence classification scores. To find optimized trained weighted values in the model, we developed preprocessing python codes, adjusted dynamic learning rate, and modifying hyper parameter in the model.

**Results:**

The model training completed; we performed classifications using the validation dataset. The results, including the confusion matrix, demonstrated performance metrics: bounding box (0.390), classification (0.034), mask (0.266), Region Proposal Network (RPN) bounding box (0.341), and RPN classification (0.054). The model successfully identified lung cancer recurrence sites, which were then accurately mapped onto chest CT images to highlight areas of primary concern.

**Conclusion:**

The trained model allows clinicians to focus on lung regions where cancer recurrence is more likely, acting as a significant aid in the detection and diagnosis of lung cancer. Serving as a clinical decision support system, it offers substantial support in managing lung cancer patients.

## Introduction

Just a century ago, lung cancer was merely considered a reportable disease. Today, it is a leading cause of mortality worldwide, affecting both developed and developing countries, including the United States. Despite significant therapeutic advancements in recent decades that have improved outcomes, lung cancer continues to be the primary cause of death across all genders. A 2022 U.S. report highlights a notable improvement in the 3-year relative survival rate for locoregional stages of lung cancer, which has risen from 21% to 31%. However, lung cancer still accounts for an average of 350 daily fatalities in the United States alone [1,2].

Lung cancer is primarily divided into two categories based on differences in clinical presentation, metastatic behavior, and treatment response: small cell (SCLC) and non-small cell lung cancer (NSCLC) [3]. NSCLC accounts for approximately 84% of all primary lung cancers, while SCLC comprises about 13% [4]. For operable stages of NSCLC, surgery aimed at complete microscopic removal of the tumor (R0 resection) remains the preferred treatment method. The standard surgical procedure involves radical pulmonary resection accompanied by mediastinal lymph node dissection. This approach not only facilitates accurate pathological staging but also informs decisions regarding subsequent adjuvant therapy [5].

Postoperative surveillance is crucial for the early detection of non-small cell lung cancer (NSCLC) recurrence, which occurs at a high rate of 30–55%, forming a vital part of clinical management. Despite adherence to the tumor-node-metastasis (TNM) staging system and National Comprehensive Cancer Network (NCCN) guidelines [6], pinpointing characteristics indicative of a high recurrence risk remains challenging. This issue has prompted numerous studies aiming to identify at-risk groups for timely interventions [7,8].

Predictive factors for recurrence include pathologic characteristics such as histologic type, tumor grade, lymphovascular invasion, and pathologic stage, alongside clinical parameters like age, sex, and serum carcinoembryonic antigen (CEA) levels [9–13]. Radiologic characteristics, notably the maximum standardized uptake value (SUVmax) from PET and CT scans, also play a pivotal role [14].

Traditional statistical analysis, while effective for data structure understanding and hypothesis validation, faces limitations with complex issues, evidenced by conflicting findings on procedures like percutaneous needle biopsies in lung cancer [15,16]. Therefore, researchers have been considering more comprehensive methodologies applicable in clinical settings, and deep learning emerges as an attractive option due to its inherent advantages in handling complex data structures, high-dimensional data, nonlinear relationships, and interaction effects. Issues related to post-surgical recurrence involve multilayered dimensions of data, including patient-related variables, pathological characteristics of the tumor, surgical techniques, and initial pharmacological treatments. Additionally, the types of data involved vary, encompassing text, images, and videos, which may present limitations for traditional statistical analysis methods. This study was conceived with the belief that the deep learning approach could offer suitable answers to such complex questions.

This study explores the use of preoperative CT imaging to identify patients at high risk of recurrence, considering the potential for more targeted interventions. Despite the utility of traditional statistical models and radiomics, limitations such as inconsistent inter-reader agreement [17] and individual radiologist factors persist [18]. We aim to overcome these limitations, enhancing prediction accuracy [19].

Our approach utilizes deep learning to predict the likelihood of postoperative lung cancer recurrence, intending to integrate finally this functionality into Electronic Medical Record (EMR) systems to support physicians. This innovation could significantly impact clinical decision-making, potentially leading to more aggressive treatment strategies. This study is the first stage of the larger project focused on developing a deep learning model to predict postoperative recurrence in operable lung cancer [20].

## Materials and methods

### Datasets

In this retrospective study, we targeted patients with primary non-small cell lung cancers who underwent curative pulmonary resections from January 1, 2010, to December 31, 2018. Patient data were anonymously extracted from the Catholic Medical Center clinical data warehouse (CMC nU-CDW) at Seoul St. Mary’s Hospital, ensuring the authors could not identify individual participants during or after data collection. The dataset included demographic information, pathologic records, operative reports, chest CT imaging files, and recurrence documentation. Out of 1,883 patients diagnosed with lung cancer and treated surgically at our hospital, those with advanced-stage disease (n = 358), metastatic lung cancer (n = 176), incomplete medical records (n = 135), or who underwent diagnostic or palliative procedures (n = 327) were excluded. This study adheres to the Declaration of Helsinki (revised in 2013) and received approval from the Catholic Medical Center’s Institutional Review Board (IRB No. KC20RASI0945), with a waiver for written informed consent due to the anonymized nature of the data.

Preoperative chest CT scans for all patients, with or without contrast enhancement and in axial, coronal, and sagittal orientations, were collected. These images, interpreted by radiologists prior to treatment, were examined using the lung setting. Key images revealing tumor characteristics were selected for analysis. Using the commercial MicroDicom DICOM viewer (https://www.microdicom.com), the surgeon (MMH) processed the raw DICOM images, selecting at least two key images from each anatomical plane for tumor location annotation. The term “key image” denotes the CT scan slice that most accurately displays the tumor’s features. These selections, aimed at optimizing analysis efficiency and informing surgical decisions, were documented in Table 1.

**Table 1:** Baseline clinical status and CT image totals in training, test, and validation groups.

	Training n = 609	Test n = 80	Validation n = 182
CT imaging Recurrence No recurrence	1,353 7,717	188 1,152	366 2,436

CT; computed tomography.

In this study, we divided the dataset into training, testing, and validation subsets with a ratio of 7:1:2, respectively. The distribution of input subject groups consisted of 609 subjects for training, 80 for testing, and 182 for validation. During the neural network training phase, we utilized only the training and testing datasets. The final validation of results was conducted using the validation dataset. Identified cancer locations were delineated on these selected images and documented in Table 1.

**Fig 1:**
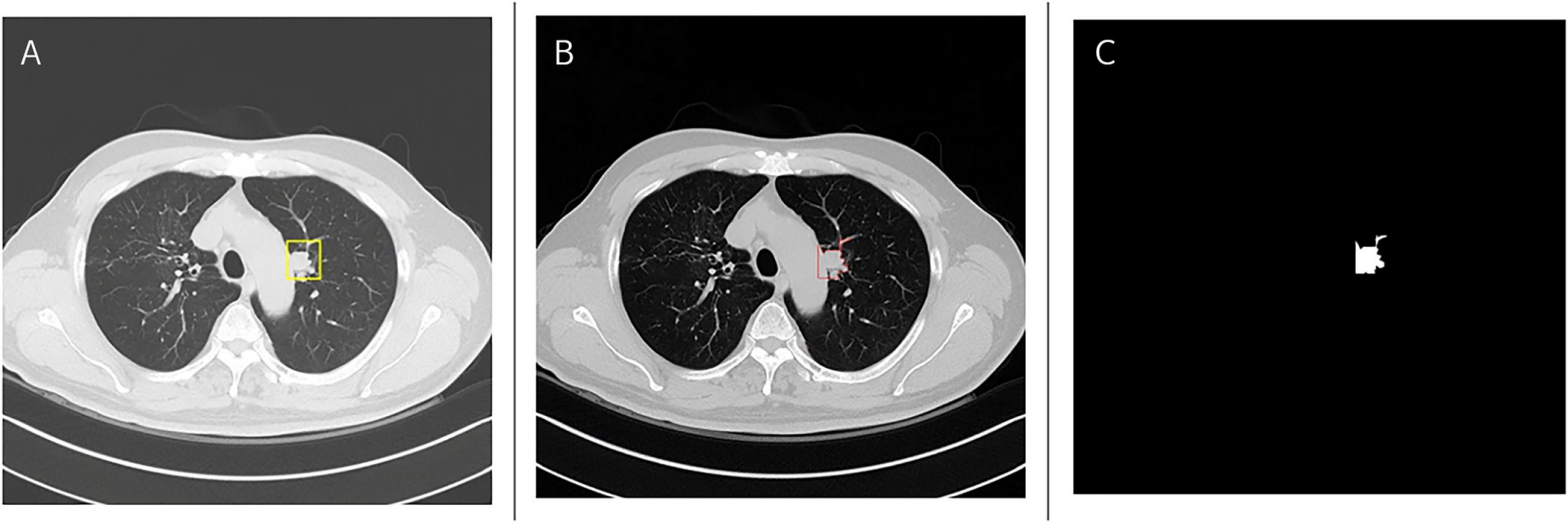
Components illustrating ground truth contours and details of a cancer site. (A) Ground truth contours; (B) Red lines indicating the shape of the cancer site after preprocessing of the annotated CT image; (C) Detailed information concerning the ground truth cancer site.

Preprocessing was performed to obtain ground truth annotations for deep learning network input data. The contour of the cancer site was derived from the resource image (Fig 1). The OpenCV package was used for preprocessing, utilizing its image manipulation functions to extract fine details from the annotated image [21]. For instance, the initial step in preprocessing involves extracting information from rectangular annotations in CT images. We utilized various OpenCV functionalities, including inRange, threshold, findContours, approxPolyDP, and fillConvexPoly. The inRange function was employed to identify areas within a specific range of color values. The combination of threshold and findContours was used to detect the contours of areas identified by inRange. ApproxPolyDP provided axis information from these contours, while fillConvexPoly was applied to create a rectangular mask. We applied these same functionalities to delineate the detailed shape of the tumor area from the annotation box, which can now be utilized with the bitwise function to highlight the tumor area in a clear and distinct shape. From this enhanced image, we can create a detailed mask of the tumor area.

### Deep learning model

To facilitate the training and visualization of lung cancer locations within CT images, we used the Mask Region-based Convolutional Neural Network (MRCNN) for deep learning purposes. This MRCNN operates through two distinct stages: the initial stage involves a region proposal network (RPN), while the subsequent stage encompasses a classification network. In the MRCNN architecture, the backbone incorporates two options for deep residual learning in image recognition, namely ResNet-101 and ResNet-50. For the specific objectives of our study, the utilization of ResNet-50 proved sufficient and appropriate. Additionally, in this study, we implemented the Mask R-CNN model, adjusting the hyperparameters disclosed in previous research to suit the analysis of lung CT images. During the training process, the learning rate was progressively adjusted in four stages: step 20, step 50, step 80, and step 120, to ensure optimal convergence of the results [22,23].

**Fig 2:**
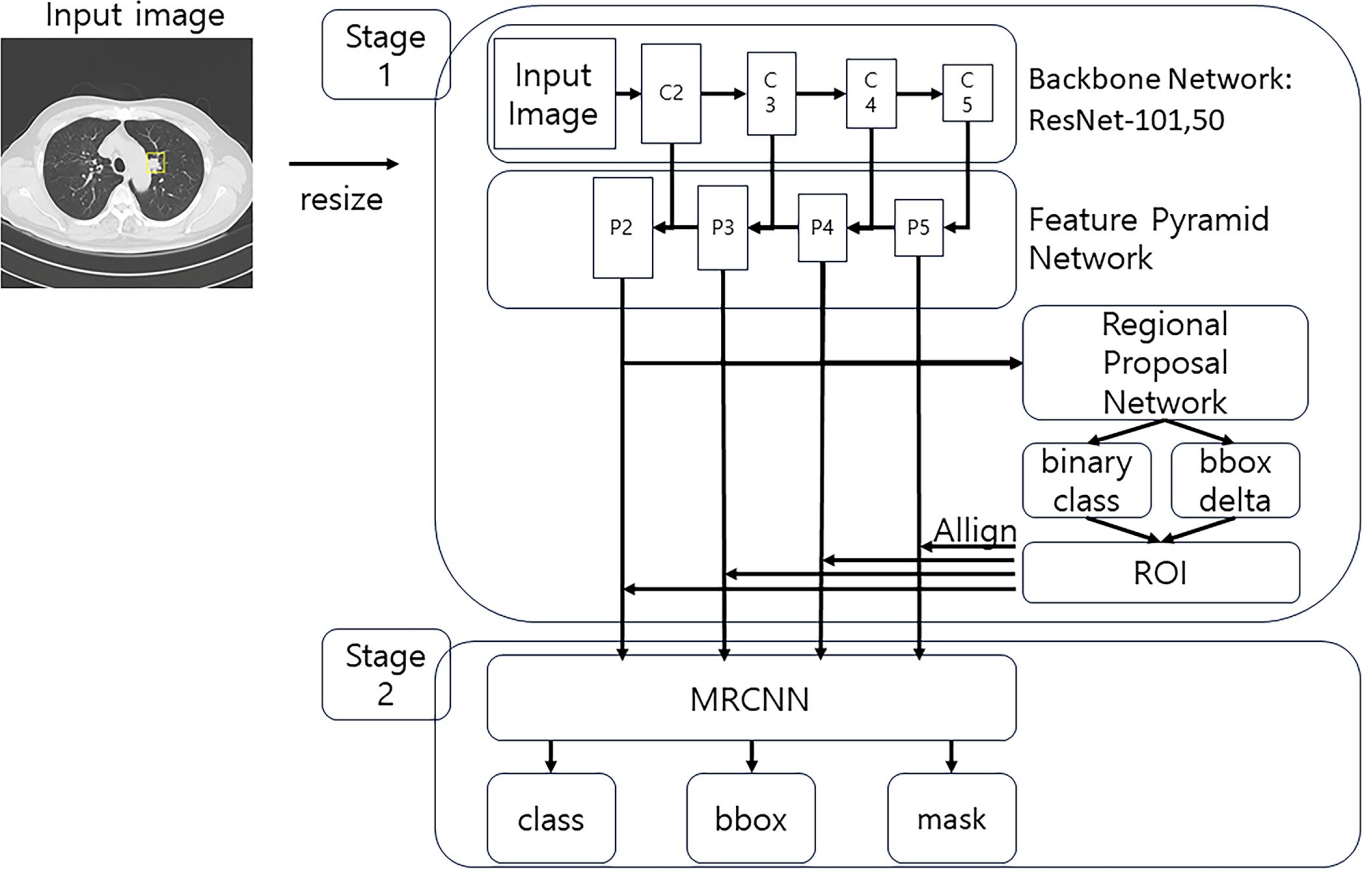
Schematic representation of the Mask Region-based Convolutional Neural Network (MRCNN) architecture in its deep learning model.

The process of generating region proposals and classification scores in an MRCNN follows a sequential workflow (Fig 2). Initially, a source image with a resolution of 1024 x 1024 undergoes appropriate scaling within the ResNet, serving as the backbone network. Subsequently, the ResNet produces five distinct feature maps: C1, C2, C3, C4, and C5. Utilizing these feature maps as input, the feature pyramid network (FPN) generates four representations denoted as P2, P3, P4, and P5 [24]. Notably, as C1 represents the resized input image, it is excluded from FPN input. The resulting feature pyramids are then utilized by the RPN network to generate ’region proposals,’ each associated with classification scores and bounding box (’bbox’) regression output values. Subsequently, suspected sites undergo further refinement based on specific classification scores. This meticulous process enables the visualization of suspected cancer locations on the original CT image while providing a predictive score for recurrence. The implementation of this process involved custom visualization programming.

In the field of lung cancer research, it is rare to develop models that can accurately detect and classify lung cancer simultaneously. Typically, models are developed for either detection or classification, but not both. Our aim is to contribute valuable insights following a clinical decision support philosophy. Our model demonstrates that even if the likelihood of recurrence is low, the ability to detect and classify simultaneously provides critical information on recurrence likelihood that physicians should consider. Consequently, we have developed a model capable of performing both detection and classification simultaneously, eliminating the need for a comparative model.

### Configuring deep learning parameters

An integral component of deep learning involves configuring parameters for network training, a process that encompasses selecting an appropriate backbone network. Prior to feeding CT source images into the deep learning network, careful consideration of several configurations is necessary. In this study, the backbone network chosen was the resnet50 [23]. An ’epoch’ represents a cycle within the deep learning network training process, symbolizing 100 training sessions. Learning rates represent the intervals between trained values, commonly referred to as weights. A higher learning rate accelerates learning; however, there exists a risk of missing an optimal point. Consequently, we adjusted the learning rate over a span of 120 epochs according to the following schedule: 0.001 for epochs 0–20, 0.0001 for epochs 21–50, 0.00003 for epochs 51–80, and 0.00001 for epochs 81–120 (Table 2).

**Table 2:** Configuration of configurations of parameters of mask region-based convolutional neural network (MRCNN) model.

Parameter	Value	Description
BACKBONE	ResNet50	Backbone network
IMAGES_PER_GPU	2	GPU count for image
NUM_CLASSES	3	Background + classes (recurrence, no recurrence)
STEPS_PER_EPOCH	100	Number of training steps per epoch
DETECTION_MIN_CONFIDENCE	0.9	Skip detections with <90% confidence
LEARNING_RATE (epoch 0–20)	0.001	Learning rate
LEARNING_RATE (epoch 21–50)	0.0001	Learning rate
LEARNING_RATE (epoch 51–80)	0.00003	Learning rate
LEARNING_RATE (epoch 81–120)	0.00001	Learning rate
RPN_ANCHOR_SCALES	(32, 64, 128, 256, 512)	List of regional proposal anchor size
MEAN_PIXEL	[76.16, 76.16, 76.16]	Mean value of pixel value
FPN_CLASSIF_FC_LAYER_SIZE	512	Feature pyramid network layer size
IMAGE_MAX_DIM	512	Image max size
MAGE_MIN_DIM	512	Image min size
IMAGE_SHAPE	[512, 512, 3]	Image shape

## Results

### Model validation results

**Fig 3:**
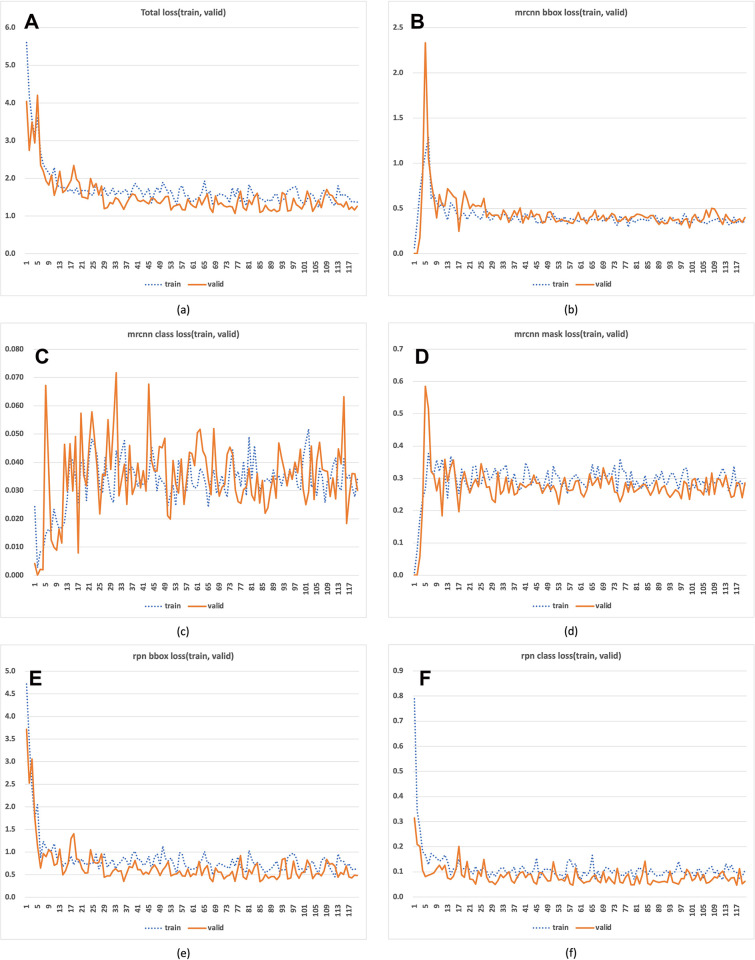
The validation results of MRCCN model. The subheadings displayed at the top of each graph, arranged from left to right, correspond to specific results of MRCNN deep learning validation loss. These are: Total validation loss (A), bounding box (B) accuracy (C), entropy loss (D), RPN bounding box loss (E), and RPN class loss (F). The ‘valid’ represents the validation set.

From the total of 120 epochs of model training, we selected the epoch that performed best based on its weight values. To evaluate model performance, we considered five loss components: total loss, MRCNN bounding box (bbox) loss, MRCNN class loss, MRCNN mask loss, RPN bbox loss, and RPN class loss, as shown in Fig 3. The total loss represents the sum of all five loss components, as indicated in Fig 3A. MRCNN bounding box loss focuses on refining the bounding box predictions within Mask R-CNN, evaluating the model’s accuracy in localizing objects within the image (Fig 3B). MRCNN class loss relates to the classifier head of Mask R-CNN, assessing the model’s accuracy in predicting class labels for detected objects (Fig 3C). MRCNN mask loss involves the binary cross-entropy loss for mask prediction in Mask R-CNN, penalizing incorrect per-pixel classifications (foreground/background) based on the true class label (Fig 3D). RPN bounding box loss deals with the regression of RPN bounding boxes, measuring the disparity between predicted and actual bounding box parameters and penalizing larger discrepancies (Fig 3E). RPN class loss pertains to the classification of anchor boxes by the Region Proposal Network (RPN) as foreground or background, evaluating the model’s effectiveness in this task (Fig 3F).

Overall, epoch 85 demonstrated the best performance, with a total validation loss of 1.085, as shown in Fig 3. This total is the sum of the bounding box (bbox) loss (0.390), classification loss (0.034), mask loss (0.266), RPN bbox loss (0.341), and RPN classification loss (0.054). In this study, we opted not to use a weighted sum of the loss components. Given the multi-task loss formulation of the MRCNN model, the aggregate of these loss components provides a comprehensive view of overall performance. Our objective was to identify a model capable of effectively detecting tumor-suspected regions while also distinguishing between recurrence and non-recurrence groups. Consequently, we were able to identify a model that exhibits a well-balanced performance in both detection and classification tasks.

### Outcome evaluation

**Fig 4:**
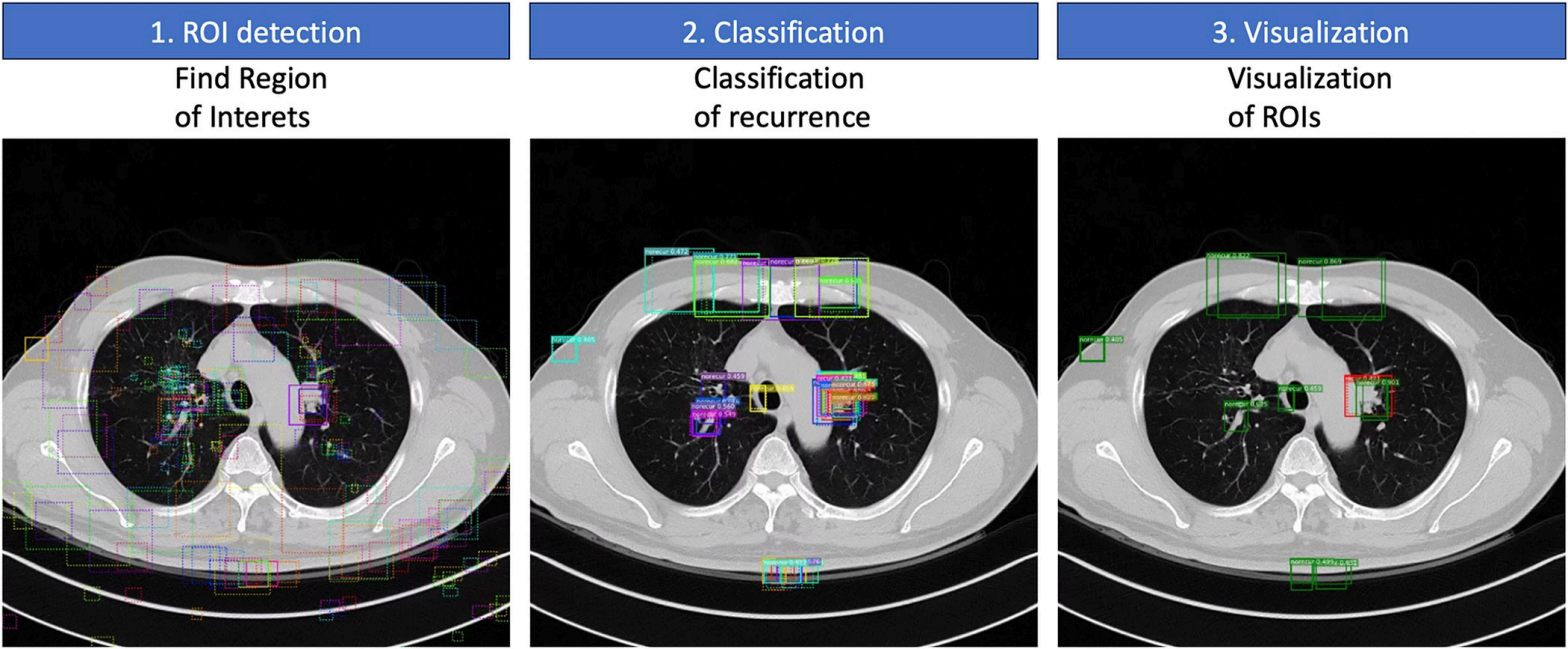
Three-step process depicting the detection of recurrence sites. From left to right: On the left, regions of interest (ROIs) generated by the region proposal network (RPN); in the center, the ROI classification step displaying each box marked to indicate the classification for recurrence and its corresponding confidence level; and on the right, the visualization showing both recurrence and no-recurrence sites".

Utilizing the MRCNN prediction model, this study entails three essential steps to detect locations and predict recurrence. Firstly, during MRCNN training, the regional proposal network (RPN) step extracts region proposals from a variety of anchor boxes covering FPN feature areas. Secondly, the proposal classification step filters classification scores above 0.3, efficiently identifying cancer sites. Finally, the visualization step showcases the recurrence site proposals alongside those lacking recurrences (Fig 4).

**Fig 5:**
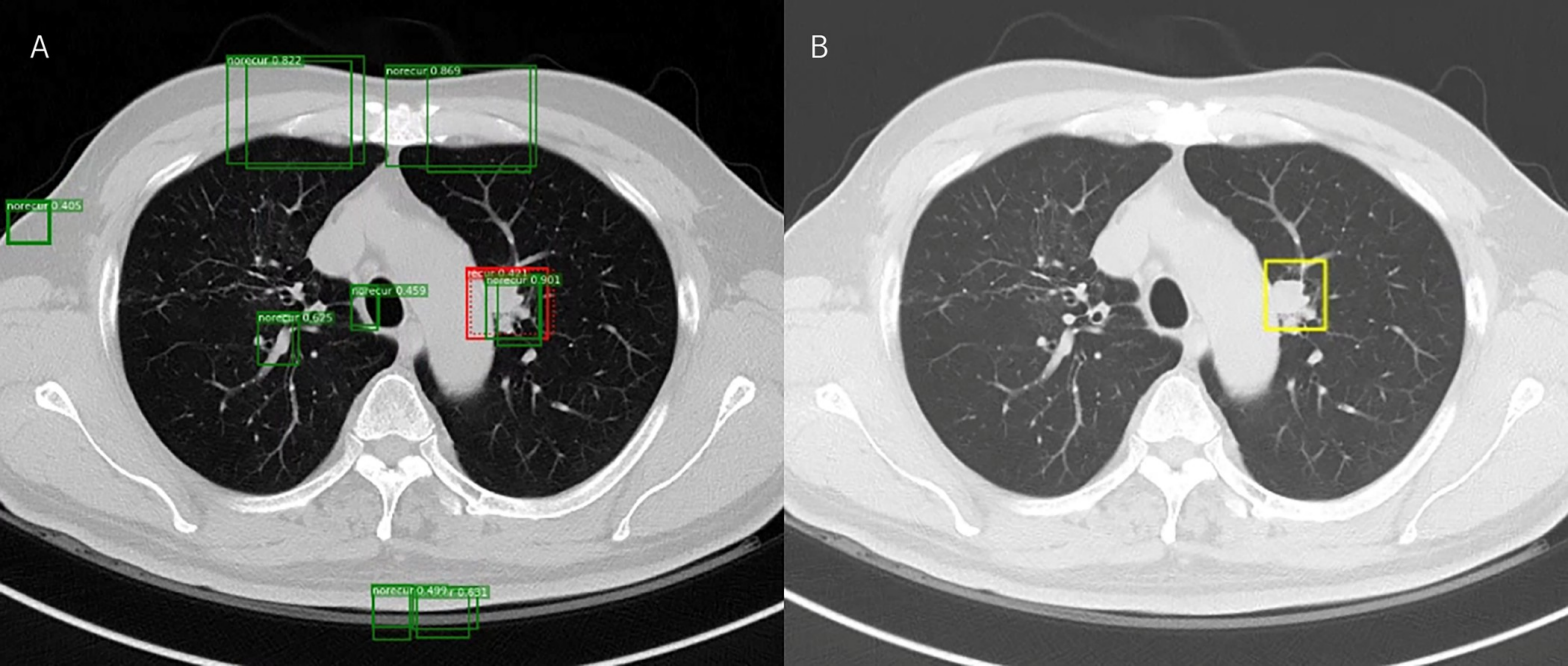
Visualization presenting recurrence sites. Recurrence sites were marked in red and no-recurrence sites were marked in green (A); annotated ground truth cancer site (B).

Physicians can utilize the CT images to visualize the predicted cancer sites. In Fig 5, the red box delineates a predicted recurrence site, while the green boxes indicate locations predicted as non-recurrence sites. The presented case was classified as a probable recurrence due to its similarity to the ground truth cancer site.

To evaluate classification performance, we analyzed the results from 182 validation subjects, comprising 163 non-recurrence subjects and 19 recurrence subjects. For physicians who require detailed information to make informed medication decisions without overlooking any possibility of recurrence, we considered a subject as having a recurrence if at least one image indicated a recurrence classification. The classification results revealed an accuracy of 0.5385, sensitivity of 0.6842, and specificity of 0.5214. The unbalanced distribution of the classification groups, coupled with the fact that each subject had multiple images yielding varied classification results, led to a high number of false positives. Fortunately, the model demonstrated high sensitivity (0.6842), correctly classifying 13 out of the 19 subjects in the recurrence group, as detailed in Table 3.

**Table 3:** Confusion matrix table of classification from mask region-based convolutional neural network (MRCNN) model.

	Reference: non-recurrence	Reference: recurrence
Prediction: non-recurrence	85	6
Prediction: recurrence	78	13

**Fig 6:**
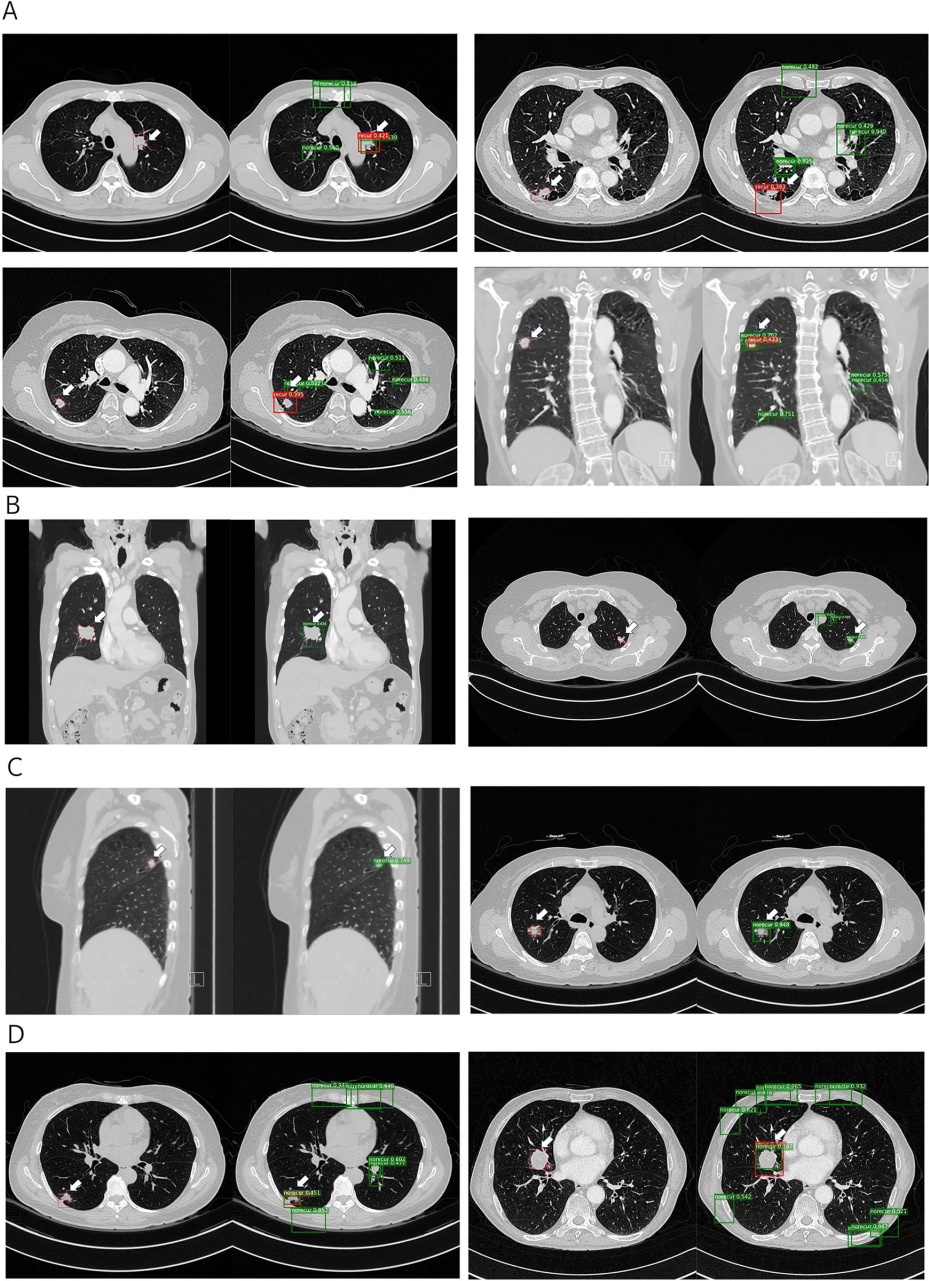
All predictive scenarios for cancer region and classification. Recurrent case classified as recurrence (A), recurrent case classified as non-recurrence (failed prediction) (B), non-recurrent case classified as no-recurrence (C), non-recurrent case classified as recurrence (failed prediction) (D).

While our model was trained using single annotated cancer sites in CT images, it holds the potential to offer diagnostic support for multiple potential cancer sites. Although there might be some noise in the detection process, the model provides classifications for recurrence sites concurrently. The various prediction permutations involved encompass scenarios such as recurrence correctly identified as recurrence, recurrence incorrectly identified as no recurrence, no recurrence correctly identified as no recurrence, and no recurrence incorrectly identified as recurrence (Fig 6).

## Discussion

### Principal results

The primary goal of the deep learning models was to support physicians in clinical decision-making by offering cancer site profiles and predicting recurrence probabilities. Initially, our findings comprised solely the maximum confidence levels of regions of interest (ROIs) derived from various prediction regions and classifications. Subsequently, we refined the results by extracting pertinent information from the deep learning model to augment the predictive data.

During training, we avoided using the weighted sum of loss components, aiming to identify a model capable of detecting cancer-suspected regions and classifying between predicted recurrence and non-recurrence groups. This strategy allowed us to select a model with balanced performance in both detection and classification.

Deep learning models, especially those applied in complex domains such as image recognition, natural language processing, and medical diagnostics, often face criticism for their lack of interpretability [25,26]. Additionally, these models encounter several issues, including a lack of transparency, difficulty in building trust, challenges in identifying biases, and obstacles in model improvement. In deep learning models with numerous layers (deep networks), the decision-making process is not transparent, making it challenging to understand how the model arrives at a specific decision, prediction, or classification. We are acutely aware of this problem and have endeavored to develop an interpretable deep learning model.

The effort to utilize deep learning for clinical decision-making began quite naturally with the advent of new technology, and research in this area is being applied throughout the entire clinical process, including tumor diagnosis, treatment decisions, and prognosis assessment [27–29]. This research represents the initial phase in a project aimed at developing an application that integrates with the hospital’s EMR system. Upon completing the algorithm’s refinement and external validation, we plan to create an application that can indicate the risk of recurrence as either high or low when a physician uploads a key image of the tumor. In this way, we aim to develop an interpretable and understandable deep learning program to aid in clinical decision-making. Should the program identify the risk of recurrence as "high" or assign specific numbers, it could lead surgeons to opt for more aggressive surgery (for example, a lobectomy rather than a segmentectomy), recommend shorter intervals of surveillance, and consider adjuvant treatment in complex cases.

### Comparable prior research efforts

Other researchers have engaged in similar endeavors to localize and classify distinct regions within magnetic resonance (MR) or CT images for deep learning models [30–33]. Previous studies have utilized either single or multiple unsupervised automated segmentation training instances as an attempt to reduce the need for labor-intensive annotations [34–36]. For instance, Yan et al. introduced a multi-instance deep learning model designed to discern body parts within CT images [32]. They developed a two-step convolutional neural network (CNN) incorporating capabilities of both a standard CNN and CNN boosting. CNN boosting integrates pre-trained local patches to enhance training, contributing to the feature pyramid network of an MRCNN model. This augmentation facilitates the identification of multiple instances within images, generating multiple feature maps.

Guo et al. also developed a deep learning model specifically tailored for the automatic segmentation of T2-weighted MRI prostate images [30]. They utilized an auto-encoder (AE) to facilitate unsupervised learning, empowering the classification layer of a supervised stacked sparse AE with hidden features. However, the primary focus of their model was to identify prostate regions within MR images rather than detecting cancerous growths.

Furthermore, Diamant et al. employed transfer learning techniques to classify chest pathology in radiographs. Their approach leveraged input features from a pre-trained CNN model to enable Support Vector Machine (SVM) functionality [31]. Through this method, the model successfully predicted pleural effusions without the need for explicit Region of Interest (ROI) delineations.

The previously mentioned studies highlight the advancement of sophisticated deep learning models through the utilization of fundamental features found in conventional methodologies like CNN and AE. Similarly, our developed MRCNN also retains profound features from its backbone network. Moving forward in this field, emphasis should be placed on targeting specific regions, particularly cancer sites, while ensuring precise classification of outcomes derived from cancer images. Our model is designed to fulfill a dual purpose: generating regions of interest (ROIs) for both localization and classification. This functionality provides invaluable support to physicians, aiding them in critical decision-making processes.

### Limitations

While projecting and visualizing cancer recurrences offer valuable advantages, it is essential to acknowledge the limitations of our model. Primarily, the model had a relatively narrow focus as it predominantly relied on training input from specific lung areas. This approach led to the identification of Regions of Interest (ROIs) confined solely to these specific areas within the original images. Consequently, this limited scope restricted the model from encompassing regions outside the lungs. However, leveraging the original image as input and processing it within our model enabled us to efficiently determine ROIs, thereby eliminating the necessity for specialized software applications.

Another limitation was the classification of lung cancer regions into recurrence and non-recurrence without definite pathological confirmation of recurrence in certain cases, leading to relatively low reliability values for the corresponding ROIs. However, despite this limitation, the accuracy of cancer predictions and the visual projections offer valuable support for decision-making. Clinical Decision Support stands as a valuable tool in enhancing surgeons’ decision-making processes by providing multiple probabilities and potential outcomes for detection and classification [18]. Our findings hold significant implications, particularly in highlighting the potential occurrence of lung cancer recurrence.

Lastly, this study has undergone internal validation; however, it lacks external validation, which limits its generalizability. The research was designed with the objective of developing a tool that could aid in CT settings and decision-making processes within our institution. Despite the absence of external validation, the dataset was divided into training, testing, and validation segments to achieve the highest possible statistical integrity. We have validated the model with validation dataset which is never used in the model training process. The research team is planning follow-up studies with the aim of creating a model capable of generalization, particularly through the inclusion of more data and, specifically, external data.

## Conclusions

The MRCNN model serves to generate multiple regions of interest and classification scores, contributing significantly to postoperative patient management for lung cancer. Aligned with Clinical Decision Support (CDS) concepts, it augments other predictive inputs with associated confidence values [18]. Physicians ultimately make decisions and may leverage deep learning to bridge information gaps. Presently, our framework offers projections of recurrence sites and classification scores, aiming to reduce potential judgment errors in future assessments.
